# Bile Duct Stenosis in a Free-Ranging Juvenile American Black Bear (*Ursus americanus*)

**DOI:** 10.3390/ani15213213

**Published:** 2025-11-05

**Authors:** Taylor Jurgens, Fern Nelson, Wesley Sheley, Alexis Johnson, Liandrie Swanepoel, Seth Wyckoff, Julie D. Sheldon

**Affiliations:** 1 Department of Small Animal Clinical Sciences, College of Veterinary Medicine, University of Tennessee, Knoxville, TN 37996, USA; tjurgens@vols.utk.edu (T.J.);; 2 Department of Biomedical & Diagnostic Sciences, College of Veterinary Medicine, University of Tennessee, Knoxville, TN 37996, USA; 3 Appalachian Bear Rescue, Townsend, TN 37882, USA

**Keywords:** bile duct, biliary outflow obstruction, bile duct stenosis, congenital, American black bear, *Ursus americanus*

## Abstract

**Simple Summary:**

This report describes the case of a juvenile American black bear that was found alone and in poor health in eastern Tennessee. The bear was taken to a rehabilitation center, where it was discovered to have abnormally high liver values on bloodwork. Despite treatment for parasites and some initial improvement in its general condition, serial blood tests revealed progressively worsening liver values. Advanced diagnostic imaging confirmed severe narrowing of the bile ducts, which deliver bile from the liver to the intestines to aid in digestion, and gallbladder dilation. Due to poor prognosis for surviving in the wild and the possibility of passing the trait to offspring if treated with surgery and released, the bear was euthanized. Necropsy confirmed the findings of biliary stenosis and suggest a possible congenital cause or less likely due to a past infection. This report is important because it is the first time such a condition has been described in a wild bear, providing new information for wildlife health experts. Understanding rare conditions like this, and how they were diagnosed, can help improve care for wild animals and support better wildlife management.

**Abstract:**

A free-ranging 10-month-old male American black bear (*Ursus americanus*) was presented to a rehabilitation facility in eastern Tennessee for being orphaned and emaciated, and was diagnosed with bile duct stenosis, leading to euthanasia. On initial intake, liver values including gamma-glutamyl transferase, alanine aminotransferase, and serum bile acids were elevated. The cub was treated for intestinal parasites and discharged to a rehabilitation facility for monitoring. Three weeks later, all liver values markedly increased despite improvements in body condition, appetite, and overall energy level. Abdominal ultrasound and dual phase computed tomography confirmed stenosis of the biliary outflow tract with gallbladder dilation and bile ducts in two locations. Surgical treatment and release were not performed due to the lack of clinical follow-up, likelihood of a genetic anomaly, and risk of it being passed to offspring. Necropsy findings further confirmed the bile duct stenosis with chronic regionally extensive fibrosis. The cause of this lesion is hypothesized to be congenital; however, inflammation secondary to a previous infection was not able to be ruled out. This case report documents the presentation and multidisciplinary approach to diagnosing a juvenile bear with bile duct stenosis, not previously reported in the literature.

## 1. Introduction

The American black bear (*Ursus americanus*) is the most widely distributed North American bear species, with populations spanning across Canada, the United States, and parts of Mexico. The Great Smoky Mountains National Park (GSMNP) is home to one of the densest black bear populations in the southeastern United States. Despite this wide range, black bears currently occupy a smaller, fragmented portion of their historical habitat due to increasing urban development, which has contributed to human–wildlife conflict [1]. Black bears are solitary, non-territorial mammals that utilize overlapping home ranges, rather than defending exclusive territories [2]. Within these ranges they are opportunistic omnivores with a seasonal dynamic diet consisting of insects, fruits, nuts, vegetation, small mammals, and carrion [3,4,5]. Human-provisioned foods have become part of the diet of bears in certain areas, including eastern Tennessee, contributing to food conditioning that may increase negative interactions with humans which poses a threat to their health [3,6]. Bears have a gastrointestinal morphology of a carnivore including short, simple intestines lacking a cecum, and the presence of a gallbladder which aids in bile storage and digestion of fats [5,7].

Morbidity and mortality of black bears in eastern Tennessee are most often related to anthropogenic causes such as vehicular trauma and human-conflict euthanasia. Cubs and yearlings may become orphaned or malnourished due to maternal mortality, vehicle strikes, illegal hunting, lack of food sources, or den abandonment during severe environmental or anthropogenic stress. On average, approximately 20 juvenile black bears are admitted each year to Appalachian Bear Rescue (ABR), a non-profit rehabilitation facility in eastern Tennessee. Veterinary care upon intake and throughout the rehabilitation process is performed through the University of Tennessee College of Veterinary Medicine (UTCVM) Zoological Medicine Service [8]. Health concerns due to primary infectious diseases are currently uncommon in this population; recent surveillance of wild bears identified exposure to and presence of several pathogens and parasites, often without clinical signs of disease [8,9]. Occasional congenital abnormalities have been identified including hydrocephalus, shoulder dysplasia, and an unidentified musculoskeletal presentation of painful and hyperflexible limb joints [8,10,11].

To the authors’ knowledge, there have been no documented cases of congenital liver disease in the free-ranging black bear population. Liver and biliary disorders that have been reported in bears are often in captivity and involve liver neoplasia or sequelae to bear bile farming in Asiatic black bears (*Ursus thibetanus*) [12,13,14]. The most common hepatic anomaly across domestic carnivore species is congenital portosystemic shunt while biliary anomalies are rare but include gall bladder agenesis and ductal plate malformation in dogs [15]. The current lack of findings in wildlife highlights the importance of wildlife health monitoring via antemortem and postmortem health assessments.

Bile is important for maintaining fat digestion; it is produced by hepatocytes and is composed of bile acids and salt, amino acids, bile pigments (bilirubin), cholesterol, electrolytes, and phospholipids [13,16]. Bile is transported to the gallbladder via the hepatic and cystic ducts where it is then stored. It then travels to the duodenum through the cystic and common bile ducts [16,17]. Any disruption in the process can cause cholestasis and further liver or intestinal disease. This report describes an emaciated 10-month-old American black bear with progressive cholestatic and hepatocellular hepatopathy, ultimately diagnosed with bile duct stenosis leading to a partial obstruction. Because the lesion was suspected to be congenital, the bear was euthanized despite improvement of clinical signs when ample food resources were provided in a rehabilitation setting. This case highlights the importance of diagnostic clinical pathology and imaging in wildlife rehabilitation medicine. 

## 2. Case Description

### 2.1. History 

A 10-month-old, 5 kg male American black bear cub (*Ursus americanus*), later designated ABR 425, was discovered alone in a tree in Greene County, TN, in November 2024, having remained in the same location for three days. The animal was presumed orphaned, captured by the Tennessee Wildlife Resources Agency and transported to the University of Tennessee Veterinary Medical Center (UTVMC) for initial examination.

### 2.2. Initial Intake Examination 

On presentation, the cub was bright, alert, and responsive. Anesthesia was induced with intramuscular xylazine (2 mg/kg, 100 mg/mL, AnaSed ^®^, VetOne, Boise, ID, USA) and ketamine (5 mg/kg, 100 mg/mL, Zetamine ^®^, VetOne, Boise, ID, USA) and maintained with isoflurane (1–5%, Fluriso®, VetOne, Boise, ID, USA) titrated via facemask. Vital parameters remained within normal limits during the 35 min procedure. For reversal, intramuscular yohimbine (0.1 mg/kg, 5 mg/mL, compounded at UTVMC Pharmacy, Knoxville, TN, USA) was administered. A physical examination, complete blood count (CBC), plasma biochemistry, urinalysis, skin scrape, and an ear skin biopsy were performed (the latter two for a sarcoptic mange surveillance study). The cub was then transferred to ABR for temporary rehabilitation.

Physical examination findings revealed emaciation with a body condition score (BCS) of 1 out of 9. The cub weighed approximately half the size of other healthy 10-month-old wild conspecifics [18]. The remainder of the exam was largely unremarkable, besides three ticks and burrs found and removed from the skin, moderate abdominal distension consistent with age, and loose feces consistent with stress. The oral cavity showed staining and moderate wear on deciduous teeth, and a loose right mandibular deciduous canine which was removed during the examination. 

Initial clinical pathology results revealed anemia (hematocrit of 28.7%, reference median 48.2% and range 37.2–53.6%), hypoalbuminemia, hypoglobulinemia, hypoproteinemia, and elevated liver enzymes including gamma-glutamyl transferase (GGT), and alanine transaminase (ALT) (Table 1) [18]. Values were compared to those from a study reporting clinical pathology values of healthy 11-month-old rehabilitated American black bears [18]. In addition, serum bile acids were mildly elevated. The remainder of the CBC and chemistry were unremarkable. This mixed hepatopathy, in addition to poor BCS, raised concern for liver disease or dysfunction, possibly due to a heavy parasitic burden such as liver flukes or *Baylisascaris* spp., or a congenital hepatic anomaly. 

Urinalysis revealed a suspected urinary tract infection with elevated red and white blood cells and struvite crystals via a free-catch sample. A routine fecal flotation using sugar and zinc floats identified the presence of *Baylisascaris* spp. eggs, likely the bear roundworm *Baylisascaris transfuga*, commonly found in this species [9]. 

The cub was treated for enteric parasites with praziquantel (34 mg; 1.5 tablets PO once), fenbendazole (100 mg/mL; 2.5 mL PO daily for three days), and amoxicillin clavulanate (62.5 mg PO BID for seven days) for the suspected urinary tract infection. He was returned to ABR with a recheck scheduled in two-three weeks, or earlier if not showing signs of clinical improvement and weight gain. During the next 18 days, the cub continued to clinically improve; he was gaining weight and eating well with normal mentation and behavior. 

### 2.3. Recheck Examination 

Recheck examination at UTCVM on day 18 occurred with the same anesthetic protocol as upon intake and revealed normal mentation, appetite, and vitals. His body weight increased to 10 kg and BCS improved to 3/9. Repeat CBC, biochemistry, and serum bile acids were performed. The complete blood count was within normal limits, and some plasma biochemical parameters improved such as albumin; however, the plasma biochemistry showed significant increases in liver and cholestatic enzymes indicative of progressive hepatic disease (Table 1). Due to persistent and progressive cholestatic enzyme elevations despite clinical improvement, abdominal imaging was pursued.

**Figure 1:**
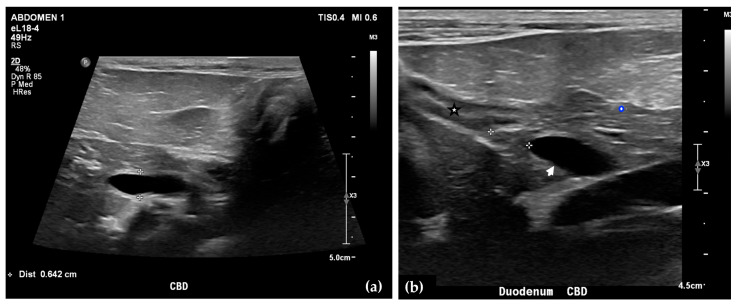
Ultrasound images of the liver. A portion of the common bile duct (CBD) is moderately-markedly distended (between + calipers) (**a**). Ultrasound image of the duodenum (black-rimmed white star), common bile duct (white arrowhead), and pancreas (blue-rimmed white circle). The distal common bile duct at the level of the duodenal papilla with abrupt narrowing (between calipers) (**b**).

**Figure 2:**
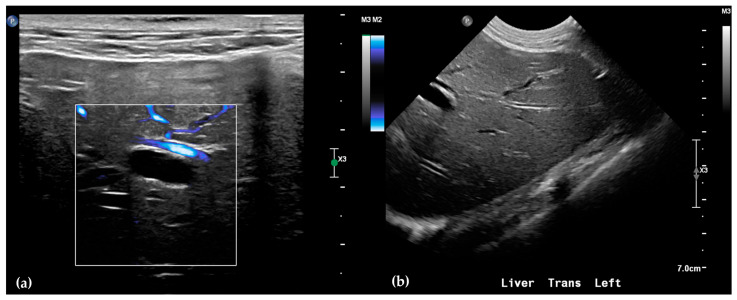
Ultrasound of the left liver. Power Doppler, within white box, of the left liver demonstrating slow flow (blue) through adjacent hepatic vasculature and no flow (black) within the anechoic structure, consistent with a dilated intrahepatic biliary duct (**a**). Several anechoic tubular structures without peripheral hyperechoic walls, consistent with peripheral intrahepatic biliary duct dilation (**b**).

Abdominal radiographs showed no significant abnormalities except a large amount of fecal material in the large intestine. The patient was placed in dorsal recumbency for an abdominal ultrasound (microconvex and linear probes 12–18 MHz, Epiq 5G, Philips Ultrasound, Bothell, WA, USA), with the ventrum shaved from above the xiphoid to the pubis in a roughly square-shaped region. Ultrasound coupling gel and alcohol were used for image optimization. The gallbladder was moderately filled with anechoic fluid and the cystic duct appropriately tapered to the common bile duct (CBD). The CBD could not be traced in its entirety, but the more distal identified segments were multifocally dilated, measuring up to 0.6 cm in diameter (outer wall to outer wall; Figure 1a). At its distal-most aspect, as it approached the duodenal papilla, the CBD abruptly tapered without a discrete intraluminal or extraluminal cause (Figure 1b). Several intrahepatic biliary ducts within the left liver hemisphere were distended distally, measuring up to 0.5 cm in dilation (inner wall to inner wall; Figure 2a,b). No intrahepatic biliary duct dilation of the right hepatic hemisphere was noted. Due to a concern for an unidentified biliary duct obstruction contributing to the patient’s clinical signs/changes, a dual phase contrast abdominal computed tomographic (CT) study was pursued for further evaluation. 

**Figure 3:**
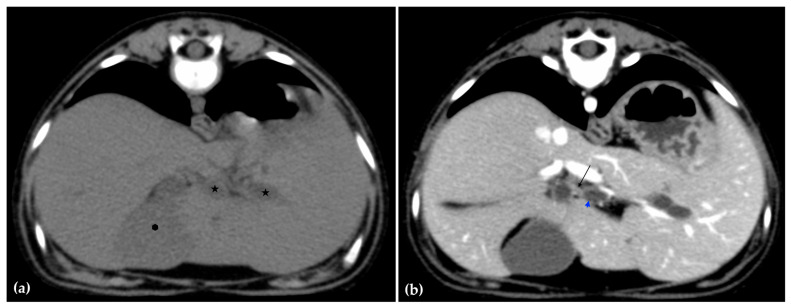
Computed tomographic (CT) pre-contrast image of the liver at the level of the gallbladder (hexagon). There is distention of the left distal intrahepatic biliary ducts (stars). The right of the patient is on the left (**a**). Computed tomographic (CT) post-contrast image (portal phase) at the level of the cystic duct (black arrow). Immediate dilation of the common bile duct (blue arrowhead) (**b**).

**Figure 4:**
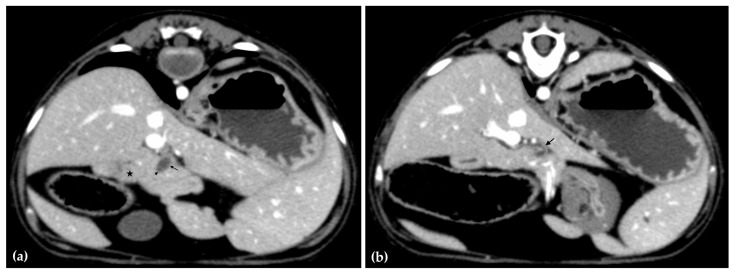
Computed tomographic (CT) post-contrast image (portal phase) at the level of the duodenal papilla (arrowhead) and duodenum (star). Focal dilation of the distal common bile duct (CBD, arrow) (**a**). Computed tomographic (CT) post-contrast image (portal phase) of the tortuous mid common bile duct (arrow) (**b**).

The patient was positioned in sternal recumbency. Helical scans of the abdomen using a multidetector CT were obtained with pre-contrast, post-contrast arterial, portal, and delayed images, all in a soft tissue reconstruction kernel (acquisition parameters: Slice thickness 0.9 mm, pitch 0.8, tube rotation time 0.5 s, 412 mA, 120 kVp, 512 × 512 matrix; Philips Brilliance-40, Philips International B.V., Amsterdam, Netherlands). Prior to contrast administration, the gallbladder was noted to be filled with predominantly mildly hyperattenuating (av. 25 HU) fluid. Dilation of the distal left intrahepatic biliary ducts were again seen (Figure 3a). Following intravenous contrast administration (Optiray 350™ Ioversol, Mallinckrodt Inc., Hazelwood, MO, USA; or Omnipaque 350™ Iohexol, GE Healthcare Inc., Marlborough, MA, USA), multifocal dilation and tortuous narrowing of the CBD was seen and best appreciated in the portal phase. There was increased conspicuity of the walls, both in contrast enhancement and wall thickness (Figure 3b and Figure 4a,b). No intraluminal debris or extraluminal structures to explain the multifocal dilation was appreciated.

Overall, abdominal ultrasound and subsequent CT imaging confirmed multifocal narrowing of the biliary outflow tract. Two regions of marked stenosis were identified: one at the junction of the common bile duct with the cystic duct, and a second at the major duodenal papilla. These findings were consistent with biliary outflow obstruction. There was no evidence of portosystemic shunting or other vascular anomalies. 

After discussion with governing wildlife agencies of a poor prognosis for release and likelihood of a congenital cause, euthanasia was performed with intravenous pentobarbital, and the bear was submitted for necropsy.

### 2.4. Necropsy 

Necropsy was performed on day 19. A standard gross necropsy examination was performed with specific focus on the hepatobiliary system. Tissues were collected and saved in 10% neutral buffered formalin. Following fixation, tissues were routinely processed for histology, paraffin-embedded, cut into 5-micron-thick sections, and stained with hematoxylin and eosin (H&E). Sections of liver were also stained with a Hall’s special stain to highlight biliary stasis, a rhodanine stain to highlight intracytoplasmic copper accumulation, and a trichrome stain to highlight fibrosis. Glass slides were digitally scanned using a Leica slide scanner, and histologic images were taken using Aperio ImageScope v12.4.6.5003.

**Figure 5:**
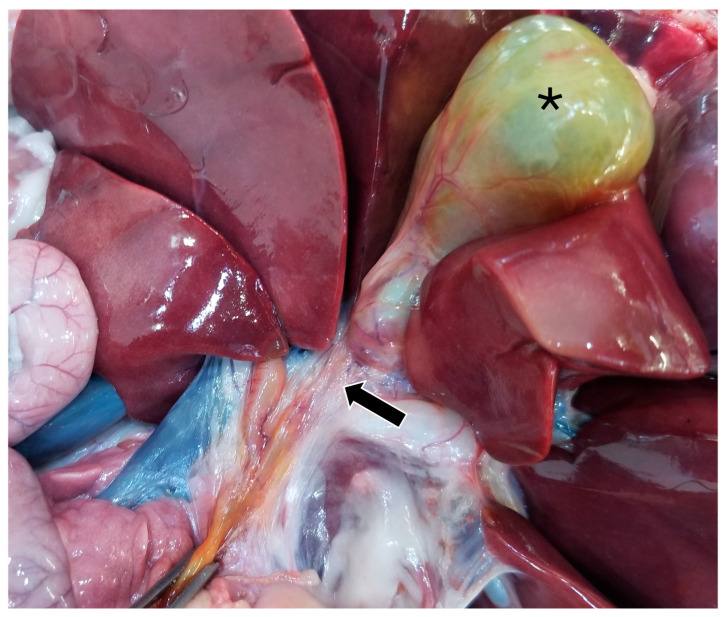
Gross image of the stenotic region of the bile duct (black arrow). The gallbladder is marked with a black asterisk.

On gross pathology, the animal was markedly small compared to what would be expected for the reported age of the animal. The liver weighed 0.38 kg (3.8% of body weight). There were numerous, multifocal to coalescing, pinpoint, pale tan foci scattered throughout all liver lobes. The bile duct was patent. The gallbladder was distended with bile and just proximal to where the cystic bile duct joins the common bile duct (Figure 5), there was a 1-cm-long abrupt narrowing of the duct with a luminal diameter of 2 mm (stenosis). The wall of the duct in the region of stenosis was thickened up to 1 mm. A 7.5 × 2.5 × 1 cm portion of the distal edge of the left lateral liver lobe was folded cranially over onto the surface of the lobe and was tightly adhered by a grey to tan, firm, thin band of tissue (fibrous adhesion).

**Figure 6:**
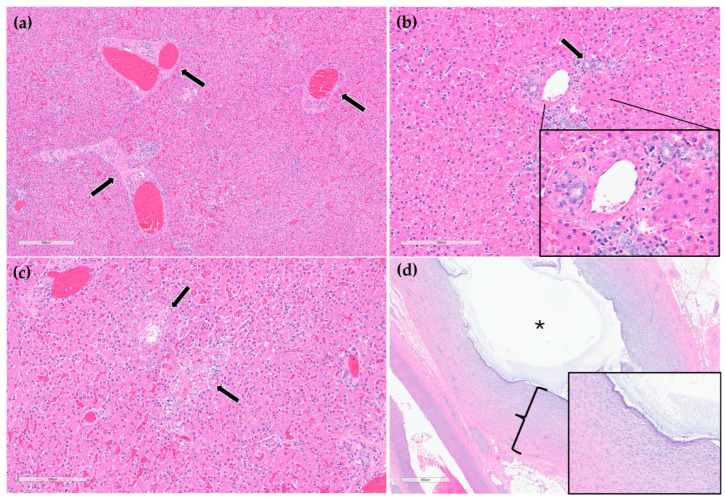
H&E stain. Example of three portal regions (black arrows) which are closely apposed and lack biliary profiles (**a**). Example of a portal region with biliary hyperplasia. Individual biliary profile pointed out with a black arrow and magnified inset (**b**). Example of multifocal, random hepatocellular necrosis (black arrows) (**c**). Stenotic bile duct. The wall is thickened by reactive fibroblasts and eosinophilic to lightly basophilic stroma (fibroplasia and fibrosis; black bracket and magnified inset). The lumen of the duct is marked with a black asterisk (**d**).

On histopathology, portal regions were variably closely apposed (up to two portal regions in a single high power (0.237 mm^2^) field) (Figure 6a). Throughout the sections of liver examined, biliary profiles were often absent or markedly decreased in size in portal regions. Occasionally, especially in areas adjacent to the gallbladder, there were increased numbers of biliary profiles in portal regions (biliary hyperplasia) (Figure 6b). Randomly scattered throughout the liver there were foci of lytic hepatocellular necrosis with an infiltrate of primarily neutrophils and fewer eosinophils (Figure 6c). Small to moderate numbers of eosinophils and neutrophils also multifocally infiltrated portal regions. The epithelium lining the cystic bile duct was variably attenuated. The wall of the cystic bile duct at the region of stenosis noted grossly was expanded by a proliferation of fibrous connective tissue with plump, reactive fibroblasts in a myxomatous matrix (fibroplasia) (Figure 6d). The sections of common bile duct examined were within normal limits. There was no evidence of either copper accumulation or bile stasis in the sections of liver stained with rhodanine and Hall’s special stains.

## 3. Discussion

This case describes a suspected congenital bile duct stenosis in a 10-month-old American black bear. The cub was presented in a severely emaciated state with evidence of liver dysfunction, most notably a severe elevation in GGT, which is a sensitive indicator of biliary disease. Despite targeted parasiticide treatment and marked improvement in body condition over a three-week period when exposed to easily accessed ample food, progressive increases in GGT, ALP, ALT, AST, and total bilirubin signified worsening hepatic injury and impaired biliary function. 

Initial concerns for hepatic parasitism or portosystemic shunting were ruled out via fecal flotation, and serum bile acids testing and imaging, respectively. Abdominal ultrasonography revealed structural abnormalities consistent with biliary outflow obstruction, including bile duct and gallbladder dilation with no visible mechanical blockage. These findings were further substantiated by CT imaging, which confirmed multifocal narrowing of the biliary tract, specifically at the hepatic-cystic duct junction and the level of the major duodenal papilla. Since bear liver anatomy does not dramatically differ from small animals or humans, minus details of lobation, these imaging features of the hepatobiliary system would not significantly differ had this case been a dog, cat or human.

Postmortem histopathologic examination of the cystic bile duct revealed fibrosis and fibroplasia of the wall leading to regionally extensive stenosis (Figure 5 and Figure 6d). This finding is suspected to have contributed to the clinical evidence of biliary obstruction. The lack of significant inflammation may be suggestive of a congenital biliary malformation rather than a secondary reactive change to inflammatory biliary disease. Portal regions in some areas of the liver were closely apposed and lacked biliary profiles which may also be supportive of a congenital anomaly (Figure 6a). With closely apposed portal regions, ductal plate malformation and congenital hepatic fibrosis were considered; however, the lack of significant periportal or bridging fibrosis, absence of portal vein hypoplasia, and absence of irregular or cystic ductal structures in this case make these less likely. Nonetheless, a mild or early form of this condition cannot be ruled out [19]. 

Other potential causes of stenosis such as a chronic, resolved inflammatory process (such as previous parasitic migration) or trauma cannot be definitively ruled out. There was no evidence of infectious organisms (bacteria, parasitic or fungal elements, or viral inclusions) in the sections of liver or biliary duct examined histologically. However, as light microscopy is often an insensitive method for identifying infectious agents, they cannot be fully ruled out using histology alone. Testing for infectious organisms (such as serology and PCR) without any clinical antemortem or postmortem evidence of infectious disease would be extremely challenging without clues of what to test for; finances were also limited. Culture of liver and bile, or bile duct tissue was not pursued but may have helped to rule out a fungal or bacterial etiology but may not directly relate to these lesions. No specific cause for the multifocal random hepatocellular necrosis was identified (Figure 6c). Considerations included acute sepsis or viral infection; however, no source of sepsis or clinical signs related to sepsis were identified. Moreover, the young age of the bear and lack of systemic signs of chronic inflammation along with lack of significant associated inflammation in the stenotic region further strengthen the suspicion for a congenital structural defect [20].

Bile duct stenosis or narrowing of the biliary ducts is a congenital anomaly affecting the liver that can severely compromise hepatic bile flow [17]. The biliary system: hepatic ducts, cystic duct, common bile duct, and gallbladder, are vital for digestion and excretion of lipophilic waste products and xenobiotics [17]. Bile synthesized in the liver is stored in the gallbladder and released into the duodenum through the major duodenal papilla during digestion. In carnivores, the flow is regulated by the sphincter of Oddi, which prevents duodenal contents from refluxing into the biliary tract [15,16]. When bile flow is obstructed, either intra- or extrahepatically, bile backs up into the liver, causing hepatocellular damage, cholestasis, jaundice, and ultimately liver failure. Causes of biliary obstruction in domestic carnivores include pancreatitis, cholelithiasis, neoplasia, trauma, or congenital malformations such as biliary atresia or stenosis and portosystemic shunts [17,19,21,22]. Clinical indicators include hyperbilirubinemia and/or marked elevations in liver enzymes such as GGT, alkaline phosphatase (ALP), ALT, and AST (aspartate aminotransferase) [15,23]. Chronic obstruction can lead to cirrhosis, portal hypertension and acquired portosystemic shunting [24]. In domestic animals, treatment of bile duct stenosis typically requires surgical bypass or ductal reconstruction, interventions that were not appropriate in this case in a free-ranging wild bear due to lack of follow-up and release considerations [25,26]. 

Congenital hepatobiliary disease in wild bear populations has not been previously documented, highlighting the importance of this case representing a novel presentation of biliary disorder in a wild American black bear. The current literature on hepatic disease in bears report that captive aging bears are affected by hepatic neoplasia and Asiatic bears are significantly affected by biliary diseases secondary to bear bile farming [12,27,28,29,30,31]. Additionally, regarding other nondomestic carnivores, two zoo-housed Amur tiger cubs were diagnosed with a congenital liver disease, but of different pathophysiology compared to this bear. The cubs had biliary ductal plate malformation and secondary acquired shunts with portal hypertension. These cubs presented systemically ill and neurologic due to hepatic encephalopathy, but, similar to this bear, hepatic enzymes progressively increased over time. Histopathological findings were characterized by portal vein hypoperfusion, not stenosis of the bile ducts themselves [32].

Although the cub showed transient clinical improvement following admission, the progressive nature of the biochemical abnormalities and confirmed anatomic malformation rendered long-term rehabilitation and release infeasible. A possible explanation for the clinical improvement while at ABR was having a controlled environment with consistent food intake, allowing the body to get out of a severe caloric deficit due to malabsorption of fats and fat-soluble vitamins from the obstruction of bile flow [16,33,34]. A stenosis that is still patent would allow for some bile to reach the small intestines and with a constant food source may allow for absorption of some nutrients and thus weight gain. However, living with a biliary flow obstruction would be insufficient for optimal digestion in the wild where food is scarce and significant energy is expended to obtain food and survive. Besides surgery, dietary changes are another less effective treatment option for managing biliary disorders in domestic animals [17]. This includes monitoring and controlling dietary fiber, omega-3, fiber, amino acids, and vitamin D that is not realistic in a wild animal [34,35]. Thus, the decision for humane euthanasia was made based on the irreversible nature of the pathology, the poor prognosis for survival, and the risk of passing this abnormality on to offspring if breeding were to occur, albeit unlikely due to lack of health.

## 4. Conclusions

To the authors’ knowledge, this is a novel case of suspected congenital bile duct stenosis in a free-ranging juvenile American black bear. Regionally extensive stenosis of the bile duct was confirmed through multimodal diagnostic imaging and histopathology. Histopathology findings supported the stenosis to be of congenital etiology due to lack of chronic inflammation in a young bear. This report highlights the importance of clinical pathology, advanced imaging and histopathology in diagnosing wildlife diseases and rare congenital anomalies, emphasizing the limitations of relying solely on physical exam in wildlife species. Furthermore, this case emphasizes the importance of wildlife health monitoring for identifying less-common diseases—both antemortem via health assessments or rehabilitation medicine and postmortem via pathology and supportive diagnostics.

## Figures and Tables

**Table 1:** Plasma biochemistry and serum bile acid results from intake (day 0) and recheck (day 18), along with reference values of healthy 11-month-old American black bears for comparison [18] when available. Abnormal (high) values are in **bold**.

Plasma Chemistry	Day 0	Day 18	Reference Median (Min–Max)
Blood urea nitrogen (BUN) mg/dL	21	24	21 (11–38)
Creatinine mg/dL	1.3	0.4	1.1 (0.8–1.3)
Total protein g/dL	4.5	7.0	6.9 (6–8)
Albumin g/dL	2.1	3.8	3.8 (3.5–4.3)
Globulin g/dL	2.3	3.2	2.9 (2.4–4.2)
A/G Ratio	0.9	1.2	1.3 (0.90–1.6)
Glucose mg/dL	127	127	164 (132–200)
Calcium mg/dL	8.5	10	8.9 (6.2–9.9)
Phosphorus mg/dL	3.8	6.4	6.6 (3–7.9)
Alkaline phosphatase (ALP) μ/L	56	**316**	84 (42–291)
Alanine Transaminase (ALT) μ/L	**160**	**462**	35 (22–100)
Aspartate aminotransferase (AST) μ/L	249	**792**	87 (52–271)
Total bilirubin mg/dL	0.2	0.1	-
Creatine kinase (CK) μ/L	152	362	241 (172–998)
Gamma-glutamyl transferase (GGT) μ/L	**1045**	**2414**	15 (5–38)
Cholesterol mg/dL	147	299	254 (208–313)
Magnesium mmol/L	1.1	0.9	1 (0.8–1.1)
Iron μg/dL	188	141	-
**Serum**			
Bile acids μmol/L	**43.7**	**77.7**	

## Data Availability

The data presented in this study are available on request from the corresponding author due to medical record privacy reasons.
